# In vitro phenotypic characterisation of two genotype I African swine fever viruses with genomic deletion isolated from Sardinian wild boars

**DOI:** 10.1186/s13567-024-01332-8

**Published:** 2024-06-07

**Authors:** Giulia Franzoni, Mariangela S. Fiori, Lorena Mura, Tania Carta, Antonello Di Nardo, Matteo Floris, Luca Ferretti, Susanna Zinellu, Pier Paolo Angioi, Anna Maria Sechi, Francesca Carusillo, Diego Brundu, Manlio Fadda, Riccardo Bazzardi, Monica Giammarioli, Stefano Cappai, Silvia Dei Giudici, Annalisa Oggiano

**Affiliations:** 1 Istituto Zooprofilattico Sperimentale Della Sardegna, 07100 Sassari, Italy; 2 Department of Veterinary Medicine, University of Sassari, 07100 Sassari, Italy; 3 The Pirbright Institute, Ash Road, Pirbright, Woking, Surrey GU24 0NF UK; 4 Department of Biomedical Sciences, University of Sassari, 07100 Sassari, Italy; 5 Pandemic Sciences Institute and Big Data Institute, Nuffield Department of Medicine, University of Oxford, Oxford, OX1 4BH UK; 6 National Swine Fever Laboratory, Istituto Zooprofilattico Sperimentale Dell’Umbria e Delle Marche, 06126 Perugia, Italy

**Keywords:** African swine fever virus, wild boar, macrophages, cytokines, genomic deletion, sequencing

## Abstract

African swine fever virus (ASFV) causes a devastating disease affecting domestic and wild pigs. ASF was first introduced in Sardinia in 1978 and until 2019 only genotype I isolates were identified. A remarkable genetic stability of Sardinian ASFV isolates was described, nevertheless in 2019 two wild boar isolates with a sustained genomic deletion (4342 base pairs) were identified (7303WB/19, 7212WB/19). In this study, we therefore performed in vitro experiments with monocyte-derived macrophages (moMФ) to unravel the phenotypic characteristics of these deleted viruses. Both 7303WB/19 and 7212WB/19 presented a lower growth kinetic in moMФ compared to virulent Sardinian 26544/OG10, using either a high (1) or a low (0.01) multiplicity of infection (MOI). In addition, flow cytometric analysis showed that both 7303WB/19 and 7212WB/19 presented lower intracellular levels of both early and late ASFV proteins. We subsequently investigated whether deleted virus variants were previously circulating in wild boars in Sardinia. In the four years preceding the last genotype I isolation (February 2015–January 2019), other eight wild boar isolates were collected, all belonging to p72 genotype I, B602L subgroup X, but none of them presented a sustained genomic deletion. Overall, we observed the deleted virus isolates in Sardinia only in 2019, at the end of a strong eradication campaign, and our data suggest that it might possess an attenuated phenotype in vivo. A better understanding of ASFV evolution in endemic territories might contribute to development of effective control measures against ASF.

**Supplementary Information:**

The online version contains supplementary material available at 10.1186/s13567-024-01332-8.

## Introduction

African swine fever (ASF) is a haemorrhagic infectious disease of suids, which is threatening the swine industry worldwide [1, 2]. The disease is spreading fast around the world; it is currently present in Africa, Europe, Asia and Oceania, with more recent outbreaks reported in the Americas [3, 4]. There is no effective vaccine against ASFV, and transmission can be prevented only by early detection and the strict application of classical disease control methods [1, 5]. The aetiological agent is African swine fever virus (ASFV), a large DNA virus belonging to the *Asfarviridae* family. This virus encodes 150–165 proteins, many of them involved in virus assembly, DNA replication and repair. In addition, ASFV encodes several proteins involved in immune-modulation, in particular evasion of host defences [1, 2]. This virus mainly replicates in cells of the myeloid lineage cells, especially monocytes/macrophage, and virulent isolates possess several strategies to counteract host responses, in order to replicate efficiently in these cells [2, 5].

The first notification of ASFV in the Mediterranean island of Sardinia dates back to 1978, and since then virus transmission was observed within and between three swine populations (domestic pigs, wild boar and illegal free-ranging pigs) in a typical biological cycle [6]. Several eradication activities against ASF in the three target populations were carried out within the framework of the last ASF regional eradication plan (PE-ASF15-18; Regional Decree Number 5/6, 6 February 2015, and subsequent additions), which strongly reduced the occurrence of the disease in the island [7, 8]. All Sardinian ASFV isolates collected from 1978 to 2019 belong to genotype I (ASF Virus Archive, Virology, Istituto Zooprofilattico Sperimentale (IZS) of Sardinia), whereas genotype II was only recently introduced (September 2023) [9]. Regarding genotype I, outbreaks in domestic pigs were detected until 2018, with the last PCR^+^ sample in wild boar dates back to early 2019 [8, 9].

Previous studies reported a remarkable stability of Sardinian ASFV genotype I isolates collected from the three swine populations [10, 11]. Despite of that, we have more recently reported on the isolation of two ASFV isolates (7303WB/19 and 7212WB/19) with a relative large genomic deletion in Sardinian wild boar in January 2019 [12]. In both viruses, a deletion of a segment of 4342 base pairs located close to the 5’ end of the genome was observed, which was never described before in the island [12].

Within this work, we aimed to unravel the phenotypic characteristics of the deleted viral isolates and therefore we carried out in vitro experiments on porcine macrophages. Subsequently, we investigated whether deleted variants were previously present in Sardinia, thus ASFV circulation in wild boar populations during the four years before the last genotype I isolation (February 2015–January 2019) was analysed in detail. The data generated in this study will extend our knowledge on ASFV evolution in this Mediterranean island, where the disease has been endemic for more than 40 years.

## Materials and methods

### Ethical statement

For in vitro experiments, blood samples were collected from healthy donor pigs. Animal husbandry, handling, and blood sampling were performed following the Italian Legislative Decree n. 26 dated 4^th^ of March 2014 and authorized by the Italian Ministry of Health (authorization n° 1232/2020-PR).

For field data, wild boar samples were collected in the framework of the PE-ASF15-18 (and subsequent additions) regional eradication plan. No wild boar was harmed or killed for this study, thus approval of the ethics committee was not required. Active surveillance was carried out mainly during the recreational hunting activities (November to January), whereas passive surveillance was achieved by inspecting reported carcasses.

### Animals for in vitro experiments

Five cross-bred pigs (*Sus scrofa domesticus*) either male or female, 6–18 months old, were used as blood donors for in vitro experiments. Animals were housed at the Experimental Station of the Istituto Zooprofilattico Sperimentale (IZS) of Sardinia (“Surigheddu”, Sassari, Italy). Animal health status was routinely monitored by trained veterinarians. The pathogens-free status was evaluated testing blood samples by both qualitative real-time PCR (African swine fever, porcine parvovirus, and porcine circovirus 2) and commercial real-time PCR kits (porcine reproductive and respiratory syndrome virus with LSI VetMAX™ PRRSV EU/NA, and *Mycoplasma hyopneumoniae* with VetMAX™-Plus qPCR Master Mix, both Thermo Fisher Scientific), following manufacturer’s instructions [13–16].

### Detection of ASFV genome in wild boar samples and virus isolation

The presence of ASFV genome in wild boar organs were investigated by real time PCR, as previously described [17, 18]. In PCR^+^ samples, the presence of infectious ASFV was assessed, using the Malmquist test, in accordance with the WOAH Terrestrial Manual [17]. In brief, tissue homogenates were added to monocytes/macrophage monolayers, which were monitored daily for haemoadsorption. In case of haemoadsorption, culture supernatant was collected and stored at −80 °C. In the absence of haemoadsorption, the test was repeated by adding culture supernatants into fresh, two-day-old monocytes/macrophage monolayers. After three negative results, absence of live ASFV virus was proved [17].

### Virus isolates and production of viral stocks

The two deleted wild boar isolates (7303WB/19, 7212WB/19) were used in the in vitro experiments, alongside a Sardinian high virulent strain (26544/OG10) used as control. 26544/OG10 (from an ASF outbreaks in Sardinia in 2010, ASF Virus Archive, Virology, IZS of Sardinia, Sassari, Italy) is regarded as a highly virulent genotype I strain: intramuscular inoculation with only 10 TCID_50_ of 26544/OG10 led to death in domestic pigs after 10–14 days (Petrini and Feliziani, unpublished data). In selected experiments, three non-deleted wild boar isolates were also used (19155WB/15, 33262WB/15, 34403WB/17).

A total of ten wild boar isolates (19155WB/15, 33262WB/15, 71926WB/15, 28784WB/16, 87152WB/16, 87326WB/16, 33747WB/15, 34403WB/17, 7303WB/19, 7212WB/19) were isolated in Sardinia between February 2015 and January 2019. Genomic analyses were carried out on six of these isolates (19155WB/15, 33262WB/15, 71926WB/15, 28784WB/16, 87152WB/16, 87326WB/16), whereas the other four (33747WB/15, 34403WB/17, 7303WB/19, 7212WB/19) were fully sequenced in our previous research works [11, 12]. Three of these isolates were also fully sequenced in this study (19155WB/15, 33262WB/15, 28784WB/16).

ASF viruses were propagated in vitro by inoculation of sub-confluent monolayers of two-day-old monocyte/macrophage cultures, in accordance with the WOAH Terrestrial Manual, using a 25 cm^2^ flask (Corning, Corning, NY, USA) [7, 17]. After incubation at 37 °C in 5% CO_2_ for two or three days, and when a clear haemoadsorbing and cytopathic effect was observed, supernatants were collected and pooled with a freeze-thawed cell lysate. The resultant pool was centrifuged at 3000 × *g* for 15 min (min) to remove debris, divided into aliquots, and stored at −80 °C. Mock-virus supernatants were prepared in an identical manner from uninfected two-day-old monocytes/macrophage cultures. Viral titers were obtained by serial ten-fold dilutions of virus suspensions on two-days-old monocyte/macrophage cultures (using 96-well plates). In details, each dilution of viral suspension was distributed to eight replicates (wells) per column (50 µL/well). After five days, cells were investigated for the presence of “rosette” (haemoadsorbing effect) and then viral titers were determined using the Spearman–Kärber formula [7, 17].

### Generation of porcine monocyte-derived macrophages

Porcine monocyte-derived macrophages (moMФ) cultures were generated from blood leukocytes, as previously described [7, 19]. In brief, leukocytes were isolated from heparinized blood and then were seeded in Petri dishes using culture media (RPMI-1640 supplemented with 10% fetal bovine serum (FBS), 100 U/mL penicillin, and 100 μg/mL streptomycin) (complete RPMI, cRPMI), supplemented with 50 ng/mL of recombinant human M-CSF (hM-CSF) (Thermo Fisher Scientific, Waltham, MA, USA) [7]. After seven days, adherent cells (moMФ) were detached by gentle scraping using a 25 mL pipette. Cells were then centrifuged at 200 × *g* for 8 min, supernatants were removed, and moMΦ counted and seeded in 12-well plates (Greiner CELLSTAR, Sigma-Aldrich, Saint Louis, MO, USA), at a density 1 × 10^6^ live cells per well. After seeding, moMφ were cultured for 24 h in un-supplemented fresh cRPMI at 37 °C 5% CO_2_ before infection [7, 19].

### Growth kinetics of ASFV in MoMφ

MoMφ were cultured in 12-well plates and 24 h later were infected with the virulent 26544/OG10 or five Sardinian ASF wild boar isolates collected between 2015 to 2019 (7303WB/19, 7212WB/19, 19155WB/15, 33262WB/15, 34403WB/17), alongside mock-infected controls. Two different multiplicity of infection (MOI) were investigated: a low (= 0.01) or a high (= 1) MOI. Virus inoculum was removed after 90 min incubation at 37 °C 5% CO_2_, then cells were washed with un-supplemented RPMI-1640 medium, and fresh cRPMI was added to the wells. MoMФ were subsequently cultured at 37 °C 5% CO_2_, and culture supernatants were collected at 0, 24, 48, and 72 h post-infection (hpi). Culture supernatants were clarified from cellular debris by centrifugation at 2000 × *g* for 3 min and then stored at −80 °C until analysis. Infectious virus levels in culture supernatants were assessed by titration, as described above. In details, each replicate was titrated independently on 2-day-old monocytes/macrophage monolayers. For each replicate, serial ten-fold dilutions of virus suspensions were prepared and each dilution was applied to eight replicates (wells) per column. After 5 days, the viral titre of each replicate was determined using the Spearman–Kärber formula [7].

### Flow cytometry

MoMφ were cultured in 12-well plates and after 24 h culture medium was removed, and cells were infected with 26544/OG10 or the deleted wild boar isolates under investigation (7303WB/19, 7212WB/19), at a MOI of 1. Mock-infected controls were included in every experiment. After 90 min incubation at 37 °C 5% CO_2_, virus inoculum was removed, cells were washed with un-supplemented RPMI-1640 medium, and fresh cRPMI was added to the wells [7]. At 24 hpi, intracellular levels of ASFV viral protein (p30 or P72) were assessed by flow cytometry, as previously described with slight modification [7, 19]. In details, cells were harvested with ice-cold PBS with 10 mM EDTA (30 min incubation at 4 °C), transferred into 5 mL round bottom tubes (Corning), fixed and permeabilized using Leucoperm, following the manufacturer’s instructions (Bio-Rad Antibodies). Intracellular levels of ASFV proteins were assessed using either anti-p72-FITC (18BG3, Ingenasa) or p30-FITC (kindly provided by Dr Francesco Feliziani, IZSUM, Italy). Cells were analyzed with a FACS Celesta (BD Biosciences) and data analysis carried out using BD FACS Diva Software (BD Biosciences), by the exclusion of doublets, gating on viable moMΦ, and then assessing the staining for intracellular viral proteins. Gates for early (p30) and late (p72) ASFV proteins were set using the mock-infected controls, as previously described [7, 19].

### Cytokine quantification

MoMφ were cultured in 12-well plates and after 24 h culture medium was removed, and cells were infected with 26544/OG10 or the deleted 7303WB/19 ASFV, alongside mock-infected controls. After 90 min incubation at 37 °C 5% CO_2_, virus inoculum was removed, cells were washed with un-supplemented RPMI-1640 medium, and fresh cRPMI was added to the wells. MoMφ stimulated with IFN-γ and LPS (both at 100 ng/mL) were used as positive control. At 24 hpi, culture supernatants were collected, centrifuged (at 2500 × *g* for 3 min) and kept at −80 °C until analysed. Levels of IL-1α, IL-1β, IL-6, IL-10, IL-12, IL-18, and TNF were determined using the Porcine Cytokine/Chemokine Magnetic Bead Panel Multiplex assay (Merck Millipore, Darmstadt, Germany) and a Bioplex MAGPIX Multiplex Reader (BioRad, Hercules, CA, USA), as previously described [19].

### Data analysis

In vitro experiments were performed in technical duplicate and repeated with at least three different blood donor pigs (biological replicates). First, baseline data distribution was evaluated based on Shapiro–Wilk test, then data were graphically and statistically analyzed with GraphPad Prism 10.01 (GraphPad Software Inc., La Jolla, CA, USA). Cytokines’ data were analysed using an un-paired T-test or the non-parametric Mann–Whitney test, all the other data were analysed using the non-parametric Kruskal–Wallis test; *p* value lower than 0.05 were regarded as statistically significant (* *p* < 0.05; ** *p* < 0.01; *** *p* < 0.001).

### DNA extraction, quantification, PCR assay and Sanger sequencing

Viral DNA was extracted from macrophage culture supernatant using a QIAmp UltraSens Virus Kit (Qiagen, Hilden, Germany), according to the manufacturer’s instructions. DNA was then quantified using an Epoch microplate spectrophotometer (BioTek) and a Qubit 3.0 Fluorometer (Thermo Fisher Scientific), following manufacturer’s instructions. Different PCRs were set up for molecular analysis [12, 20]. In details, 71926WB/15, 87152WB/16, 87326WB/16 samples were genotyped by sequencing (Sanger) a fragment of the B646L gene (p72) and the B602L gene (CVR), using primers and thermal cycling conditions previously described [20]. Furthermore, in these isolates the presence of deletion of 4342 base pairs near the 5’ was assessed using a set of specific primers (reported in Additional file 1) and a PCR protocol previously described [12]. Three isolates were fully sequenced (19155WB/15, 33262WB/15, 28784WB/16, described below), their sequences of the B602L and EP402R genes were confirmed by Sanger sequencing using primers and the methods that were described previously [20].

### Genome sequencing

Three wild boar isolates where fully sequenced in this study: 19155WB/15, 33262WB/15, 28784WB/16. Viral libraries were generated using Nextera DNA Flex Library Prep kit (Illumina Inc., San Diego, CA, USA) starting from a minimum DNA input of 50 ng, according to manufacturer’s protocols, as previously described [12]. Library size profiles had an average fragment size of 600 bp. Sequencing was carried at AMES Group (Centro Polidiagnostico Strumentale S.r.l, Napoli, Italy) out on Novaseq 6000 (Illumina) diluting libraries to the starting concentration for the system. A median coverage of 250 was obtained [12, 21–23].

Genome data processing was performed using an in house bioinformatic pipeline. The bcl2fastq program [23] was used to convert BCL files generated to standard FASTQ file formats. Trim Galore [24] was used to quality trim the data and remove sequencing adaptors. These reads were then aligned to the pig reference genome (Sus scrofa 10.2 [25]) using the bwa-mem algorithm [26]. Aligned bam files were then sorted and indexed with samtools [27] and deduplicated with Picard-tools [28]. To obtain high-quality variants, freebayes [29] was used to call variants for each sample, using the KX354450.1 sequence as reference genome (parameters: “--ploidy 1 -X -u -m 20 -q 20 -F 0.2”).

### Phylogenetic analyses

To investigate the evolutionary relationship of wild boar isolates collected between 2015 and 2019 among the historical context of the ASFV genotype I in Sardinia, a phylogenetic analysis was performed using an alignment consisting of (i) 78WGSs generated from viruses isolated in the island between 1978 and 2019 (including 3 wild boar isolates fully sequenced in this study) and (ii) 3 WGSs from viruses of European and African origins. A maximum-likelihood phylogeny was reconstructed in IQ-TREE 2.2.0 [30] by modeling the nucleotide substitution with a general time-reversible (GTR + Γ) model with empirical base frequencies. The obtained phylogeny was then time-calibrated using TreeTime 0.11.1 [31] (Additional file 2).

## Results

### In vitro characterization of wild boar isolates

In vitro assays were performed to phenotypically characterize the deleted wild boar viruses isolated in January 2019. Porcine macrophages represent the main target of ASFV, in which the virus replicates efficiently [32]. We therefore assessed the ability of the deleted isolates to infect and replicate in these cells. The two deleted wild boar isolates (7303WB/19 and 7212WB/19) were compared with the Sardinian virulent strain 26544/OG10.

**Figure 1:**
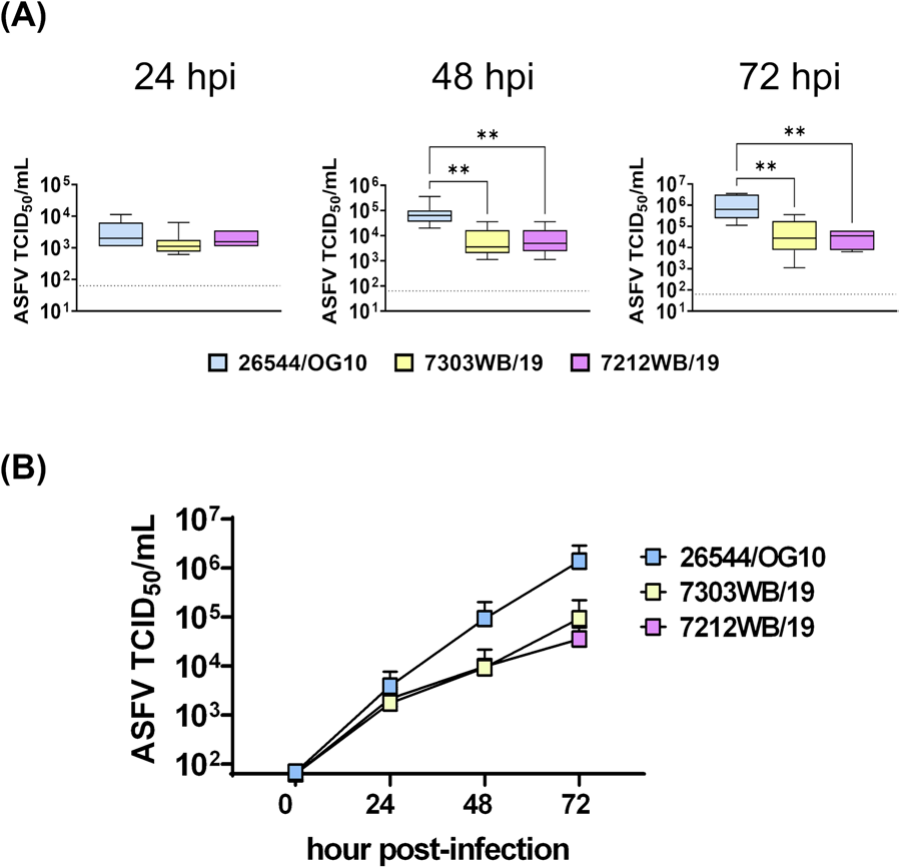
**Growth kinetic in macrophages of Sardinian wild boar ASFV isolates (MOI = 0.01).** MoMФ were infected with 7303WB/19, 7212WB/19, or the virulent 26544/OG10, using a MOI of 0.01. Samples were collected in duplicate at 24, 48, and 72 hpi and infectious viral progeny in culture supernatants were assessed by titration (TCID_50_/mL). The sensitivity of this assay was 1.8 log_10_ 50% haemadsorbing doses/mL (TCID_50_/mL). Data from four biological replicates are presented. In panel **A**, data of 7303WB/19 and 7212WB/19 were compared to those of the virulent 26544/OG10 using the non-parametric Kruskal–Wallis; ** *p* < 0.01. In panel **B**, viral growth curves are presented.

A kinetic analysis of the infection with these viruses in moMФ was performed first using a MOI of 0.01. Culture supernatants were collected at different time post-infection (0, 24, 48, and 72 hpi) and viral titers were determined by titration. Both Sardinian isolates with a deletion of 4342 base pairs near the 5’ (7303WB/19 and 7212WB/19) replicated less efficiently in these cells, with lower levels of infectious virus in culture supernatants at 48 and 72 hpi (Figure 1).

**Figure 2:**
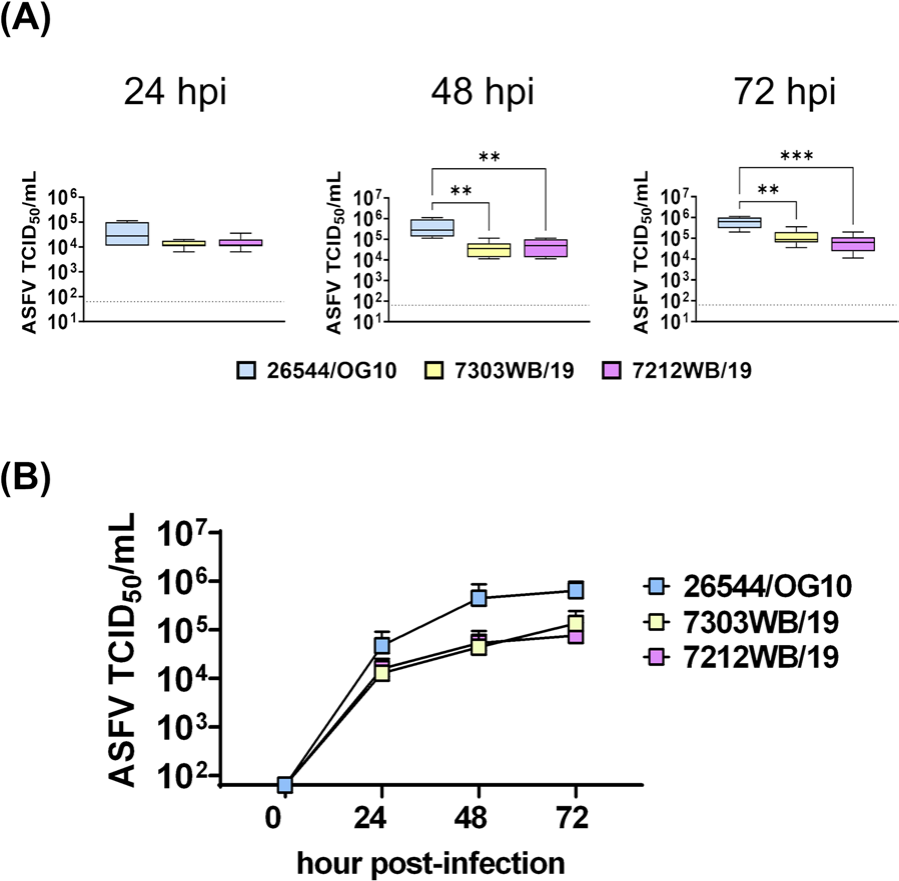
**Growth kinetic in macrophages of Sardinian wild boar ASFV isolates (MOI = 1).** Samples were collected in duplicate at 24, 48, and 72 hpi and infectious viral progeny in culture supernatants were assessed by titration (TCID_50_/mL). The sensitivity of this assay was 1.8 log_10_ 50% haemadsorbing doses/mL (TCID_50_/mL). Data from four biological replicated are presented. In panel **A**, results obtained from the wild boar isolates were compared to those of the virulent 26544/OG10 using the non-parametric Kruskal–Wallis; ** *p* < 0.01; *** *p* < 0.001. ﻿ In panel **B**, viral growth curves are presented.

Then, we investigated whether these differences were still appreciable using a higher MOI. MoMФ were infected with the same viruses using a MOI of 1 at 0, 24, 48 and 72 hpi and viral levels in culture supernatants were determined by titration. Lower levels of infectious viral particles were observed in the culture supernatants of two deleted wild boar isolates (7212WB/19 and 7303WB/19) compared to virulent 26544/OG10, at both 48 and 72 hpi (Figure 2).

**Figure 3:**
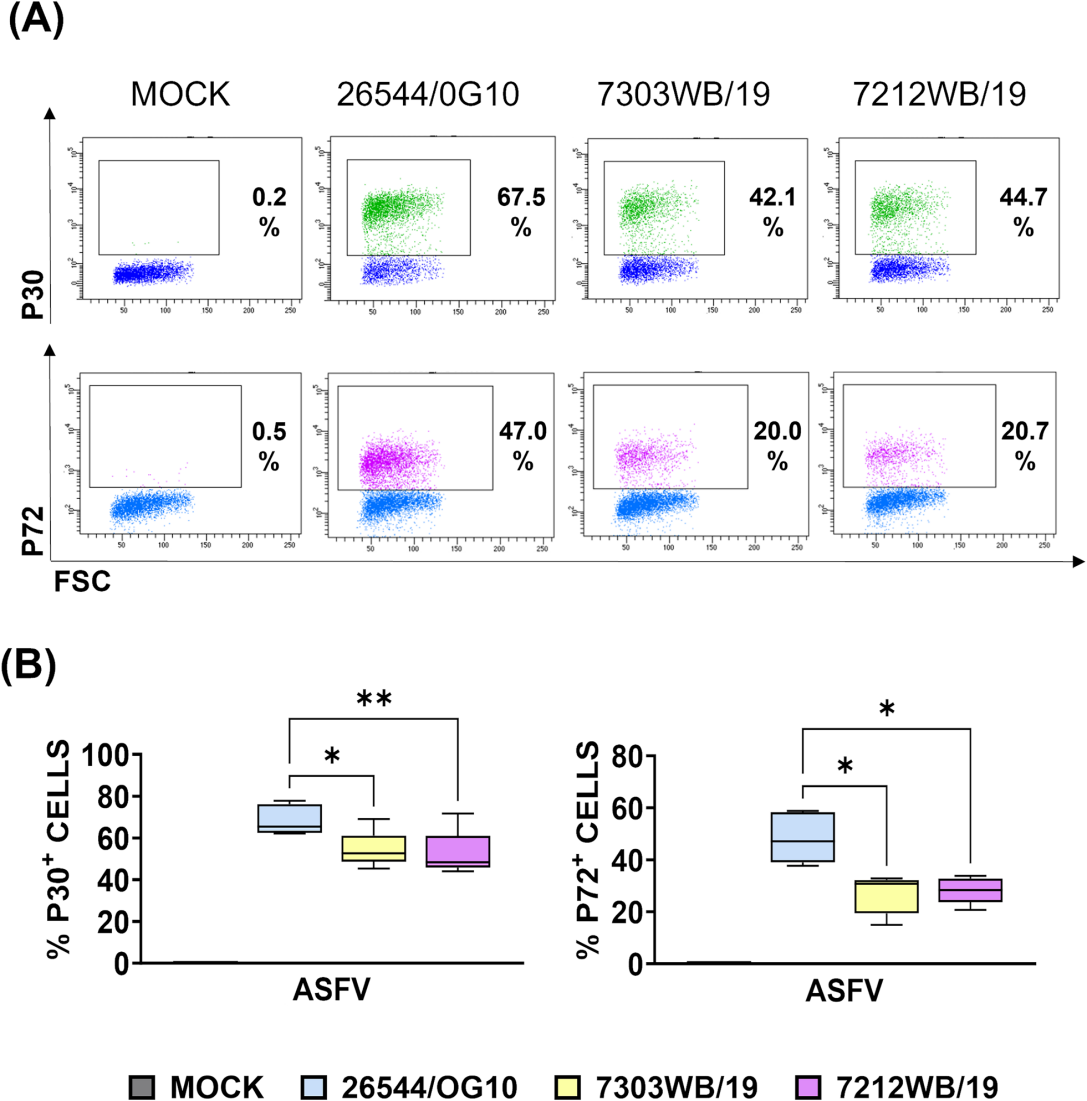
**Intracellular levels of ASFV viral protein in moMФ infected Sardinian wild boar ASFV isolates.** MoMФ were infected with either the wild boar isolates under study or the virulent 26544/OG10, using a MOI of 1. Mock-infected moMФ were used as control. 24 h later, intracellular levels of ASFV viral protein were analyzed by flow cytometry. **A** Representative dot plots; **B** box and whisker plots illustrate intracellular levels of ASFV early protein P30 and late protein P72. Data from four biological replicated are presented. 7303WB/19 and 7212WB/19 data were compared to those of the virulent 26544/OG10 using the non-parametric Kruskal–Wallis; * *p* < 0.05; ** *p* < 0.01.

Subsequently, flow cytometry was employed to determine differences between the viruses in the intracellular levels of ASFV viral proteins. MoMФ were infected using a MOI of 1 and 24 h later intracellular levels of ASFV viral proteins (early protein P30 and late protein P72) were determined using flow cytometry. At 48 and 72 hpi the vast majority of ASFV-infected moMФ were detached, so it was impossible to carry on flow cytometric analysis in those samples. Lower levels of both early P30 and late P72 were observed in the two deleted wild boar isolates (7212WB/19 and 7303WB/19) compared to virulent 26544/OG10 at 24 hpi (Figure 3).

**Figure 4:**
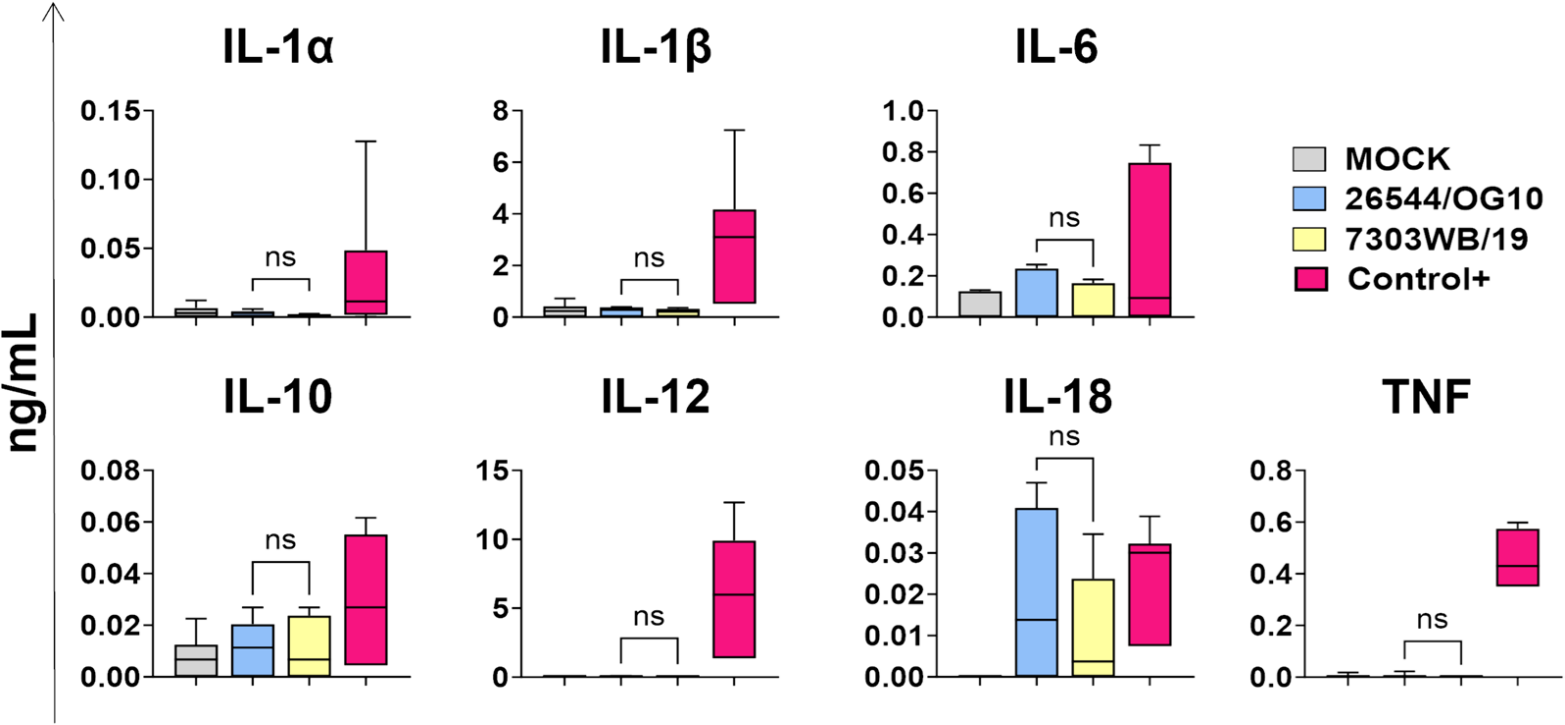
**Cytokines release in response to infection.** MoMФ were infected with either the deleted 7303WB/19 or the virulent 26544/OG10, using a MOI of 1. Mock-infected moMФ were used as negative control, moMФ stimulated with IFN-γ + LPS (both at 100 ng/mL) were used as positive control (Control+). 24 h later, cytokines’ levels in culture supernatants were analysed by milliplex ELISA. Data from three biological replicates are presented. For each cytokine, 7303WB/19 and 26544/OG10 data were compared using un-paired T test or a Mann-Whitey test.

It is widely known that virulent ASF viruses developed several mechanisms to evade host immune responses, such as inhibition of cytokines induction/release by infected macrophages [32]. We therefore investigated whether infection with the deleted isolate triggered release of pro-inflammatory cytokines. MoMФ were infected with 7303WB/19 or 26544/OG10 using a MOI of 1, alongside mock-infected controls. 24 hpi, cytokine contents in culture supernatants was assessed. We observed that neither the deleted 7303WB/19 or the virulent 26544/OG10 ASFV triggered release of pro-inflammatory cytokines (IL-1α, IL-1β, IL-6, IL-12, IL-18, TNF) from macrophages. No release of anti-inflammatory IL-10 was also detected (Figure 4).

### ASFV circulation in wild boar in Sardinia: ten ASFV isolates collected between February 2015 and January 2019

We subsequently investigated whether the deleted virus isolates were previously circulating in wild boar populations in Sardinia. We analysed in details wild boar samples collected the four years preceding the last genotype I isolation (February 2015 and January 2019). In this time-frame, 18 247 wild boars were tested for the presence of ASFV, with ASFV genome detected in 76 animals (Additional file 3). PCR^+^ samples had frequently high Ct values, suggesting presence of low levels of viral genome in the majority of tested organs (Additional file 3). Before January 2019, virus isolation was achieved in PCR^+^ organs from eight different wild boar, all of them between February 2015 and March 2017 (19155WB/15, 71926WB/15, 33262WB/15, 33747WB/15, 87152WB/16, 87326WB/16, 28784WB/16, 34403WB/17), and none in 2018 (Table 1, Additional file 4). As expected, virus isolation was frequently achieved in PCR^+^ samples with a low Ct values (mean = 22) (Additional files 3, 5).

**Table 1:** **Genomic information of the wild boar ASFV isolates collected between February 2015 and January 2019**

Isolate	Genotype (B646L)	Subgroup (B602L)	Deletion at the left variable region	References
33747WB/15	I	X	NO	[11]
19155WB/15	I	X	NO	This study
33262WB/15	I	X	NO	This study
71926WB/15	I	X	NO	This study
87152WB/16	I	X	NO	This study
87326WB/16	I	X	NO	This study
28784WB/16	I	X	NO	This study
34403WB/17	I	X	NO	[11]
7303WB/19	I	X	YES	[12]
7212WB/19	I	X	YES	[12]

Typing analysis were carried out in six wild boar isolates under study (19155WB/15, 33262WB/15, 71926WB/15, 28784WB/16, 87152WB/16, 87326WB/16), whereas whole genome sequences of the other four wild boar isolates collected between February 2015 and January 2019 were generated in our previous research works (33747WB/15, 34403WB/17, 7303WB/19, 7212WB/19) [11, 12]. The analysis of the C-terminal end of the B646L gene (coding for p72) revealed that all wild boar isolates collected between February 2015 and January 2019 belonged to genotype I, similar to all other Sardinian ASFV isolates collected between 1978 and 2019 (Table 1, Additional file 2). Analysis of the B602L gene showed that all these isolates clustered in subgroup X (Table 1), as with almost all the Sardinian isolates collected between 1990 and 2019 (“modern” Sardinian isolates).

As above stated, we recently reported that 7303WB/19, 7212WB/19 presented a deletion of 4342 base pairs near the 5ʹ, not found in other Sardinian isolates [12], so in this study we investigated whether a similar deletion was present in Sardinian wild boar samples collected in the previous years. Whole genome sequences of the three isolates fully sequenced in this study (19155WB/15, 33262WB/15, 28784WB/16) showed that they were almost identical in size and number of open reading frame (ORF) to the other Sardinian isolates collected until 2018 [11, 12] (Additional file 6). In addition, typing analysis was carried out on the other three wild boar isolates (71926WB/15, 87152WB/16, 87326WB/16) in order to exclude the presence of the deletion. The whole region encompassing the deletion was amplified using specific primers [12], then PCR was carried out, and the amplicons’ size were assessed on 2% agarose gel. Only 7303WB/19 and 7212WB/19 ASFV presented a deletion (Table 1, Additional file 7).

**Figure 5:**
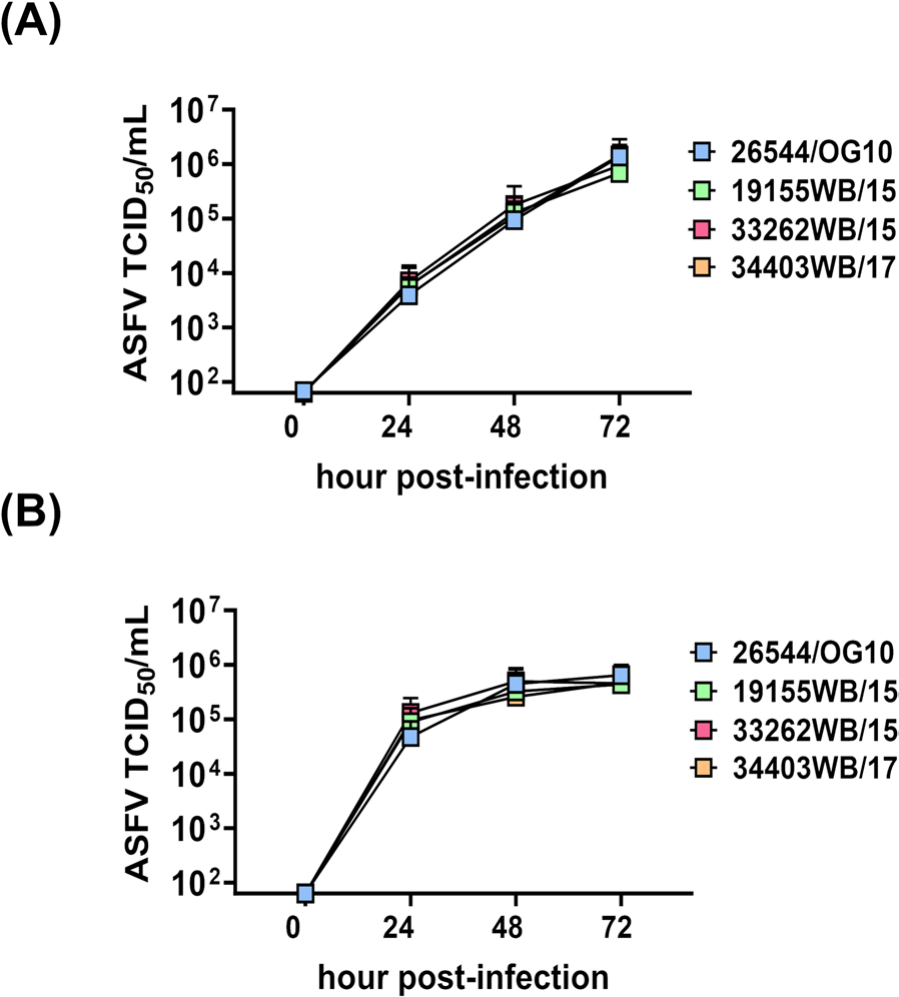
**Growth kinetic in macrophages of non-deleted Sardinian wild boar ASFV isolates.** MoMФ were infected with the virulent 26544/OG10 or three non-deleted wild boar isolates (19155WB/15, 33262WB/15, 28784WB/16), using a MOI of 0.01 (**A**) or 1 (**B**). Samples were collected in duplicate at 24, 48, and 72 hpi, and infectious viral progeny in culture supernatants were assessed by titration (TCID_50_/mL). The sensitivity of this assay was 1.8 log_10_ 50% haemadsorbing doses/mL (TCID_50_/mL). Data from four biological replicated are presented. At each time-point, results obtained from the wild boar isolates were compared to those of the virulent 26544/OG10 using the non-parametric Kruskal–Wallis.

As expected, the non-deleted ASFV wild boar isolates showed growth kinetics in macrophages similar to those of the virulent Sardinian 26544/OG10 ASFV (Figure 5).

**Figure 6:**
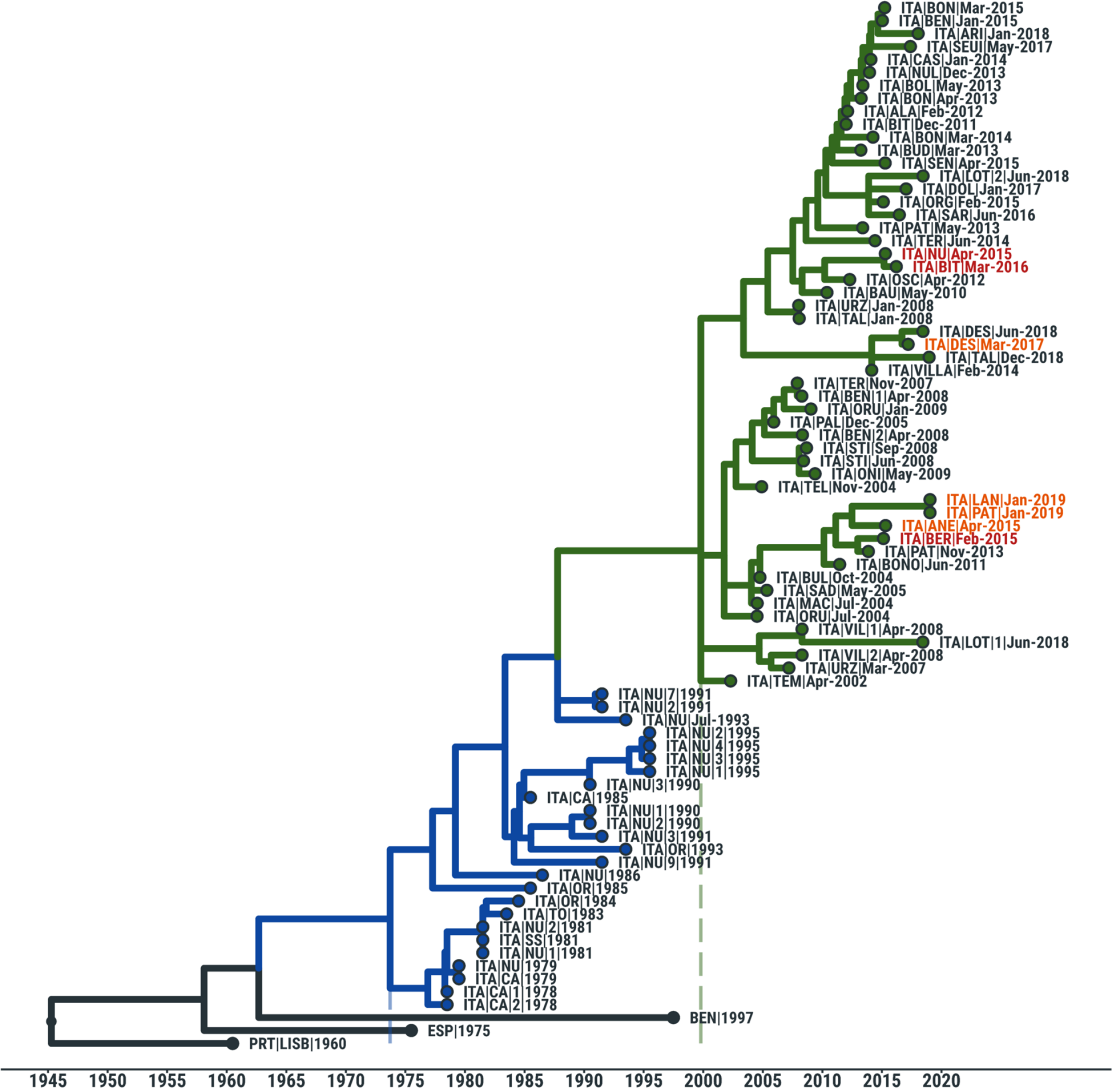
**Time-scaled phylogeny of Sardinian wild boar isolates under study.** Time-scaled phylogeny of 78 ASF viruses isolated from Sardinia between 1978 and 2019 and 3 ASF viruses collected from Europe and Africa. Coloured branches indicate phylogenetic clades that include: (i) ASF viruses collected between 1978 and 1995 (blue), (ii) and those viruses isolated after 2000 (green). ASFV sequences generated in this study (19155WB/15, 33262WB/15, 28784WB/16) are indicated in red font, whilst the other four wild boar sequences previously sequenced [11, 12] (33747WB/15, 34403WB/17, 7303WB/19, 7212WB/19) are indicated in orange font.

Then, phylogenetic analyses were performed in order to better understand the origin of the deleted ASFV isolates in Sardinia. Phylogenetic reconstruction assigned the newly sequenced ASFV isolates into two different clades, within the larger cluster of Sardinian viruses collected after 2000 (Figure 6): 19155WB/15 was assigned to a minor clade containing a few recent Sardinian sequences from 2004 to 2015, sister to the one characterizing the two ASFV isolates containing the deletion (7303WB/19 and 7212WB/19); 33262WB/15 and 28784WB/16 were found to be closely related and descending from viruses circulating within the provinces of Sassari and Nuoro during 2012 and 2010, respectively (Figure 6).

## Discussion

ASFV has been present in Sardinian since 1978 and it has been endemic for more than four decades; all the Sardinian ASF viruses isolated between 1978 and 2019 belong to genotype I. No other ASFV introductions from outside the island were notified until September 2023, when the genotype II was first reported [13]. We previously analysed ASFV circulation in Sardinian free-ranging pigs [7], which were regarded as virus reservoirs in the island, and we observed a high percentage of animals with ASFV Abs at the time of culling, despite being apparently healthy and in good nutritional status. In details, ASFV Ab were detected in 36.7% of free-ranging pigs culled between December 2017 to February 2020 (2491 tested out of 4484 culled) [7]. It is assumed that these pigs had likely recovered completely from infection, thus we investigated whether low-virulence virus variants were circulating among these animals [7]. ASFV strains with reduced virulence were indeed identified in other endemic countries, belonging to either genotype I (NH/P68 and OURT 88/3 in Portugal) [33] or II (Estonia2014, Lv17/WB/Rie1 in East Europe, Pig/Heilongjiang/HRB1/2020 in China) [34–36].

The two isolates collected from Sardinian ASFV Ab^+^ apparently healthy free-ranging pigs (55234/18 and 103917/18) did not present genomic deletions or an attenuated phenotype in vitro [7] and other studies described a remarkable genetic stability of Sardinian ASFV isolates [10, 11]. Nevertheless, two Sardinian ASFV isolates with a sustained deletion (> 4000 base pairs in length), belonging to genotype I, were isolated in wild boar in January 2019 (7303WB/19 and 7212WB/19 ASFV) [12], only few month before the last ASFV genotype I PCR^+^ samples in the island (April 2019).

We therefore assessed whether the deleted viral isolates presented a different phenotype. In vitro experiments on porcine macrophages were performed, considering that macrophages represent indeed the main target of ASFV. The virus developed several strategies to evade host defences and to replicate efficiently in these cells; these mechanisms are instead partially lost in ASFV attenuated strains [5, 32]. Our experiments revealed that the deleted 7303WB/19 and 7212WB/19 presented a diminished growth kinetic in porcine moMФ compared to the virulent Sardinian 26544/OG10 ASFV. Both 7303WB/19 and 7212WB/19 have a large deletion encompassing the genes MGF 360-6L, X69R, MGF 300-1L [12]. The deletion of one or more of these genes likely affect ASFV ability to replicate in its target cells. In the past, researchers suggested that X69R promoted virus replication [37], nevertheless a more recent study showed that X69R gene is not essential for ASFV replication in macrophages in vitro [38]. In details, it was observed that a mutant lacking X69R (ASFV-G-ΔX69R) and its parental virulent ASFV Georgia07 (ASFV-G) had a similar growth kinetic in porcine macrophages [38]. It is plausible to think that unlikely the X69R deletion alone is responsible for the growth defect observed in the two Sardinian wild boar isolates collected in 2019. 7303WB/19 and 7212WB/19’s deletion encompasses also two genes belonging to the ASFV Multigene families (MGFs), which are a group of genes located within the left terminal and right terminal of the ASFV genome [39]. Although the function of many of these genes is still unknown, it was reported that several MGF proteins are involved indifferent steps of the viral reproduction cycle and infection, including transcription, translation, virulence and immune escape [40, 41]. In particular, MGF360 and MGF505 seems to play a crucial role in evading host cells defence mechanisms, suppressing IFN-I responses [42, 43]. In addition, ASFV MGF360 and MGF530 genes are porcine macrophage host range determinants, which promote survival of ASFV-infected macrophages [44]. The lack of MGF 360-6L and MGF 300-1L might therefore be related to the macrophage growth defect of 7303WB/19 and 7212WB/19.

With the aim to further characterize the effects of this deletion, we assessed the release of key immune cytokines in response to infection. We and others previously described that virulent ASF viruses inhibited induction/release of pro-inflammatory cytokines by infected macrophages (reviewed in [32]); in this work we investigated whether the deleted 7303WB/19 differed from the virulent Sardinian 26544/OG10. Our data showed that there were no differences in the cytokine profile of macrophages infected with either Sardinian ASF viruses.

Nevertheless, we can still hypothesize that the deleted Sardinian ASFV isolates likely possess an attenuated phenotype in vivo, based on their diminished growth kinetics in porcine macrophages. Other researchers reported that several deleted ASFV mutants were characterized by both a macrophage growth defect and a reduced pathogenicity in vivo. In particular, several deleted mutants originated from the virulent genotype II ASFV Georgia07 (ASFV-G) showed both a delayed and/or diminished replication in porcine macrophages compared to the parental strain and an attenuated phenotype in vivo: ASFV-G-ΔH108R, ASFV-G-ΔA104R, ASFV-G-ΔE184L, ASFV-G-ΔA137R, ASFV-G-ΔI117L [45–49]. Future studies should investigate whether the deletion encompassing MGF 360-6L, X69R and MGF 300-1L genes resulted in an attenuated phenotype in vivo.

We subsequently investigated whether deleted virus variants were previously present in Sardinia. ASFV circulation in wild boar populations in Sardinia during the four years preceding the last genotype I isolation (February 2015–January 2019) was analysed in details. We reported that ASFV genome was detected in 76 out of 18 247 tested wild boar samples, but virus isolation was achieved only from ten animals, eight between 2015 and 2017 and none in 2018. All these ten isolates were characterized as genotype I subgroup X, similarly to all the Sardinian isolates collected between 1990 and 2019. Most importantly, a sustained deletion in the genome was not observed in all isolates collected before 2019.

Seven out of ten of these isolates (33747WB/15, 19155WB/15, 33262WB/15, 28784WB/16, 34403WB/17, 7303WB/19, 7212WB/19) were fully sequenced and phylogenetic analysis showed that those isolates evolved from different ASF viruses circulating in Sardinia since 2010, with only 33747WB/15 and 19155WB/15 isolates clustering with those ASFV isolates presenting the 4342 nt length deletion. In our previous work we hypothesized that the deletion originated with a single mutational event in the ancestral lineage of 7212WB/19 and 7303WB/19, which occurred during late 2012 [12]. With this work, we attempted to better understand its origin, nevertheless no other wild boar isolates with this deletion were detected before 2019. 7212WB/19 and 7303WB/19 sequences are separated by their ancestral clade by a relatively long branch, so we might speculate that this may be due to insufficient sampling. These deleted viruses were discovered at the end of a strong eradication campaign, so another option is that similar ASFV immune-escaped variants might existed before in Sardinia, but they were rapidly taken over by ASFV variants with a stronger ability to replicate and disseminate in the host.

Overall, in this work we analysed ASFV circulation in wild boar in Sardinia in the four years before the last genotype I isolation (January 2019). A total of ten wild boar isolates were collected in this time frame and we observed a sustained genomic deletion (>4000 base pairs) only in the two collected in January 2019 (7303WB/19, 7212WB/19). In vitro experiments revealed that only the two mutated viruses presented a lower growth kinetic in moMФ compared to the virulent Sardinian wild type 26544/OG10 ASFV, which likely results in attenuated phenotype in vivo. ASFV deleted isolates were observed in the island after 40 years of ASFV, at the end of a strong eradication campaign, suggesting that other ASFV viral variants might occurred before, but they rapidly extinguished. Further studies should assess the impact of viral variants on ASFV persistence in an endemic territory.

### Supplementary Information

**Additional file 1. List of primers used to investigate the deletion near the 5’ of ASFV.**

**Additional file 2. Metadata associated with ASFV whole genomes analysed in this study.** GenBank accession number and tip labels used in the phylogenetic trees are also reported. Samples used for the seven isolates under study are in red, four fully sequenced in our previous research works (Fiori et al., [11, 12]) and three in this study (bold text).

**Additional file 3. Information on ASFV PCR + wild boar in Sardinia between February 2015 and January 2019.** In the table information regarding geographic origin, time of collection, virological (tested organs, Ct values of real-time PCR, Malmquist) and serological (ELISA and Immunoblotting) results are reported. Samples under study are in red.

**Additional file 4. Map showing Sardinian ASFV wild boar isolates under study.** Map showing Sardinian ASFV wild boar isolates under study. The map shows the location of the 10 wild boar isolates used in this study and collected in Sardinia between February 2015 and January 2019.

**Additional file 5. Threshold cycle values of real-time PCR**^**+**^** wild boar samples.** Wild boar isolates collected in Sardinia between February 2015 and January 2019 by both passive and active surveillance. Threshold cycle values of real-time PCR^+^ wild boar samples are presented. Values of Malmquist + and Malmquist- samples were compared using a Mann–Whitney test; * *p* < 0.05, ** *p* < 0.01.

**Additional file 6. Genomic sequence of the seven fully sequenced wild boar ASFV isolates collected between February 2015 and January 2019.** Genomic sequence of the seven fully sequenced wild boar ASFV isolates collected between February 2015 and January 2019.

**Additional file 7. Agarose gel electrophoresis (2%) of PCR amplification products from five wild boar isolates under study (71926WB/15, 87152WB/16, 87326WB/16, 7303WB/19, 7212WB/19).** PCR was carried out using specific primers for the deletion near the 5’ (listed in Table S1). Lane M: size marker VIII; lanes 1: ASFV1-DEL; lanes 2: ASFV2-DEL lanes 3: ASFV4-DEL lanes 4: ASFV6-DEL lanes 5: ASFV8-DEL; lane 6: K − .

## Data Availability

In vitro data presented in the study are available on request from the corresponding author. Wild boar field data (PCR^+^ samples) are included in supplementary information. The three ASFV whole genome sequences obtained in the study are openly available in the Gen Bank nucleotide sequence database (accession numbers OP312970, ON260841, ON260840).
