# Supplementary material for: In vitro phenotypic characterisation of two genotype I African swine fever viruses with genomic deletion isolated from Sardinian wild boars
Source: Vet Res. 2024 Jun 7;55:73. doi: 10.1186/s13567-024-01332-8 (PMC11157848; doi:10.1186/s13567-024-01332-8)
Supplement: Supplementary file 1 — Additional file 1. List of primers used to investigate the deletion near the 5’ of ASFV. [file 13567_2024_1332_MOESM1_ESM.docx]

**Additional file 1. List of primers used to investigate the deletion near the 5’ of ASFV**

| **Primer sequence** | **Amplicon length** | **Reference** |
| --- | --- | --- |
| ASFV1‐DEL_F: CAGACGTTGCCTATTCGG  ASFV1‐DEL_R: GCTGAGAGACAATTTGCG | 688 | [12] |
| ASFV2‐DEL_F: CCGCAAATTGTCTCTCAG  ASFV2‐DEL_R: GCTCTGACGTTGACAGCT | 938 | [12] |
| ASFV4‐DEL_F: GCTGTCAACGTCAGAGCA  ASFV4‐DEL_R: CACCAGTGAACCTGTTTC | 330 | [12] |
| ASFV6‐DEL_F: GATGTCAGGCTTATCTGGA  ASFV6‐DEL_R: ACGGACGTTGTTATCCTGG | 820 | [12] |
| ASFV8‐DEL_F: CTGTACAGATGCTAAAGCAA ASFV8‐DEL_R: ACATATTTCACATCCGTGGC | 854 | [12] |
