# Supplementary material for: In vitro phenotypic characterisation of two genotype I African swine fever viruses with genomic deletion isolated from Sardinian wild boars
Source: Vet Res. 2024 Jun 7;55:73. doi: 10.1186/s13567-024-01332-8 (PMC11157848; doi:10.1186/s13567-024-01332-8)
Supplement: Supplementary file 2 — Additional file 2. Metadata associated with ASFV whole genomes analysed in this study. GenBank accession number and tip labels used in the phylogenetic trees are also reported. Samples used for the seven isolates under study are in red, four fully sequenced in our previous research works (Fiori et al., [11, 12]) and three in this study (bold text). [file 13567_2024_1332_MOESM2_ESM.docx]

**Additional file 2. Metadata associated with genotype I ASFV whole genomes analysed in this study.** GenBank accession number and tip labels used in the phylogenetic trees are also reported. Samples used for the seven strains under study are in red, four fully sequenced in our previous research works [11, 12] and three in this study (bold text).

| SeqID | SeqName | SampleID | GenBank | Host | Sampling | Country | City |
| --- | --- | --- | --- | --- | --- | --- | --- |
| PRT\|LISB\|1960 | PRT\|LISB\|1960 | L60 | NC044941 | Domestic Pig | 1960 | Portugal | Lisbon |
| ESP\|1975 | ESP\|1975 | E75 | NC044958 | Domestic Pig | 1975 | Spain |  |
| ITA\|CA\|1\|1978 | ITA\|CA\|1\|1978 | 56/Ca/1978 | MN270969 | Domestic Pig | 1978 | Italy | Cagliari |
| ITA\|CA\|2\|1978 | ITA\|CA\|2\|1978 | CA1978_2 | MW723480 | Domestic Pig | 1978 | Italy | Cagliari |
| ITA\|NU\|1979 | ITA\|NU\|1979 | NU1979 | MW723481 | Domestic Pig | 1979 | Italy | Nuoro |
| ITA\|CA\|1979 | ITA\|CA\|1979 | 57/Ca/1979 | MN270970 | Domestic Pig | 1979 | Italy | Cagliari |
| ITA\|SS\|1981 | ITA\|SS\|1981 | SS1981 | MW788409 | Domestic Pig | 1981 | Italy | Sassari |
| ITA\|NU\|1\|1981 | ITA\|NU\|1\|1981 | 139/Nu/1981 | MN270971 | Domestic Pig | 1981 | Italy | Sassari |
| ITA\|NU\|2\|1981 | ITA\|NU\|2\|1981 | NU1981_2 | SRR13976567 | Domestic Pig | 1981 | Italy | Nuoro |
| ITA\|TO\|1983 | ITA\|TO\|1983 | ITALY1983 | SRR13976568 | Domestic Pig | 1983 | Italy | Torino |
| ITA\|OR\|1984 | ITA\|OR\|1984 | OR1984 | MW800838 | Domestic Pig | 1984 | Italy | Oristano |
| ITA\|OR\|1985 | ITA\|OR\|1985 | 140/Or/1985 | MN270972 | Domestic Pig | 1985 | Italy | Oristano |
| ITA\|CA\|1985 | ITA\|CA\|1985 | 85/Ca/1985 | MN270973 | Domestic Pig | 1985 | Italy | Cagliari |
| ITA\|NU\|1986 | ITA\|NU\|1986 | NU1986 | MW723482 | Domestic Pig | 1986 | Italy | Nuoro |
| ITA\|NU\|1\|1990 | ITA\|NU\|1\|1990 | NU1990_1 | MW723483 | Domestic Pig | 1990 | Italy | Nuoro |
| ITA\|NU\|2\|1990 | ITA\|NU\|2\|1990 | NU1990_2 | SRR13976561 | Domestic Pig | 1990 | Italy | Nuoro |
| ITA\|NU\|3\|1990 | ITA\|NU\|3\|1990 | 141/Nu/1990 | MN270974 | Domestic Pig | 1990 | Italy | Nuoro |
| ITA\|NU\|2\|1991 | ITA\|NU\|2\|1991 | NU1991_2 | MW723484 | Domestic Pig | 1991 | Italy | Nuoro |
| ITA\|NU\|3\|1991 | ITA\|NU\|3\|1991 | NU1991_3 | MW723485 | Domestic Pig | 1991 | Italy | Nuoro |
| ITA\|NU\|7\|1991 | ITA\|NU\|7\|1991 | NU1991_7 | MW723486 | Domestic Pig | 1991 | Italy | Nuoro |
| ITA\|NU\|9\|1991 | ITA\|NU\|9\|1991 | NU1991_9 | SRR13976566 | Domestic Pig | 1991 | Italy | Nuoro |
| ITA\|NU\|1993-07-1 | ITA\|NU\|Jul-1993 | NU1993_2 | MW723488 | Domestic Pig | 01/07/93 | Italy | Nuoro |
| ITA\|OR\|1993 | ITA\|OR\|1993 | OR1993_1 | MW723487 | Domestic Pig | 1993 | Italy | Oristano |
| ITA\|NU\|1\|1995 | ITA\|NU\|1\|1995 | 142/Nu/1995 | MN270975 | Domestic Pig | 1995 | Italy | Nuoro |
| ITA\|NU\|2\|1995 | ITA\|NU\|2\|1995 | NU1995_2 | MW723489 | Domestic Pig | 1995 | Italy | Nuoro |
| ITA\|NU\|3\|1995 | ITA\|NU\|3\|1995 | NU1995_3 | MW723490 | Domestic Pig | 1995 | Italy | Nuoro |
| ITA\|NU\|4\|1995 | ITA\|NU\|4\|1995 | NU1995_4 | MW723491 | Domestic Pig | 1995 | Italy | Nuoro |
| BEN\|1997 | BEN\|1997 | BENIN_1997 | NC044956 | Domestic Pig | 1997 | Benin |  |
| ITA\|TEM\|2002-04-19 | ITA\|TEM\|Apr-2002 | 24225_2002 | MW788411 | Domestic Pig | 19/04/02 | Italy | Tempio |
| ITA\|ORU\|2004-07-7 | ITA\|ORU\|Jul-2004 | 44076_2004 | MW723500 | Domestic Pig | 07/07/04 | Italy | Orune |
| ITA\|MAC\|2004-07-12 | ITA\|MAC\|Jul-2004 | 45539_2004 | SRR13976563 | Domestic Pig | 12/07/04 | Italy | Macomer |
| ITA\|BUL\|2004-10-5 | ITA\|BUL\|Oct-2004 | 26/Ss/2004 | MN270977 | Domestic Pig | 05/10/04 | Italy | Bultei |
| ITA\|TEL\|2004-11-26 | ITA\|TEL\|Nov-2004 | 74377_2004 | MW723496 | Domestic Pig | 26/11/04 | Italy | Telti |
| ITA\|SAD\|2005-05-5 | ITA\|SAD\|May-2005 | 22649_2005 | MW723497 | Domestic Pig | 05/05/05 | Italy | Sadali |
| ITA\|PAL\|2005-12-1 | ITA\|PAL\|Dec-2005 | 72398WB_2005 | MW723495 | Wild boar | 01/12/05 | Italy | Palau |
| ITA\|TER\|2007-11-23 | ITA\|TER\|Nov-2007 | 72407_2007 | MN270978 | Domestic Pig | 23/11/07 | Italy | Tergu |
| ITA\|URZ\|2007-03-11 | ITA\|URZ\|Mar-2007 | 72912WB_2007 | MW723498 | Wild boar | 11/03/07 | Italy | Urzulei |
| ITA\|URZ\|2008-01-6 | ITA\|URZ\|Jan-2008 | 1537WB_2008 | MW788405 | Wild boar | 06/01/08 | Italy | Urzulei |
| ITA\|TAL\|2008-01-20 | ITA\|TAL\|Jan-2008 | 4996WB_2008 | MW723492 | Wild boar | 20/01/08 | Italy | Talana |
| ITA\|BEN\|2008-04-10 | ITA\|BEN\|1\|Apr-2008 | 22137_2008 | MW723499 | Domestic Pig | 10/04/08 | Italy | Benetutti |
| ITA\|VIL\|2008-04-16 | ITA\|VIL\|1\|Apr-2008 | 22943_2008 | MW788406 | Domestic Pig | 16/04/08 | Italy | Villasor |
| ITA\|VIL\|2008-04-17 | ITA\|VIL\|2\|Apr-2008 | 23221_2008 | MW723494 | Domestic Pig | 17/04/08 | Italy | Villasor |
| ITA\|BEN\|2008-04-28 | ITA\|BEN\|2\|Apr-2008 | 25185_2008 | MW788410 | Domestic Pig | 28/04/08 | Italy | Benetutti |
| ITA\|STI\|2008-06-1 | ITA\|STI\|Jun-2008 | 46830_2008 | MW723493 | Domestic Pig | 01/06/08 | Italy | Stintino |
| ITA\|STI\|2008-09-1 | ITA\|STI\|Sep-2008 | 47/Ss/2008 | KX354450 | Domestic Pig | 01/09/08 | Italy | Stintino |
| ITA\|ORU\|2009-01-15 | ITA\|ORU\|Jan-2009 | 1628_2009 | SRR14601691 | Wild boar | 15/01/19 | Italy | Orune |
| ITA\|ONI\|2009-05-22 | ITA\|ONI\|May-2009 | 28170_2009 | SRR13976565 | Domestic Pig | 22/05/09 | Italy | Oniferi |
| ITA\|BAU\|2010-05-20 | ITA\|BAU\|May-2010 | 26544/OG10 | KM102979 | Domestic Pig | 20/05/10 | Italy | Baunei |
| ITA\|BONO\|2011-06-14 | ITA\|BONO\|Jun-2011 | 31208_2011 | MW736612 | Domestic Pig | 14/06/11 | Italy | Bono |
| ITA\|ALA\|2012-02-12 | ITA\|ALA\|Feb-2012 | 2019WB_2012 | MW736598 | Wild boar | 12/02/12 | Italy | Ala deiSardi |
| ITA\|OSC\|2012-04-18 | ITA\|OSC\|Apr-2012 | 97/Ot/2012 | MN270979 | Domestic Pig | 18/04/12 | Italy | Oschiri |
| ITA\|BIT\|2011-12-30 | ITA\|BIT\|Dec-2011 | 63525WB_2011 | MW736603 | Wild boar | 30/12/11 | Italy | Bitti |
| ITA\|BUD\|2013-03-25 | ITA\|BUD\|Mar-2013 | 30322_2013 | MW736600 | Domestic Pig | 25/03/13 | Italy | Budduso |
| ITA\|BON\|2013-04-3 | ITA\|BON\|Apr-2013 | 32516_2013 | MW736607 | Domestic Pig | 03/04/13 | Italy | Bonorva |
| ITA\|PAT\|2013-05-20 | ITA\|PAT\|May-2013 | 47039_2013 | MW736597 | Domestic Pig | 20/05/13 | Italy | Pattada |
| ITA\|BOL\|2013-05-27 | ITA\|BOL\|May-2013 | 49179WB_2013 | MW736601 | Wild boar | 27/05/13 | Italy | Bolotana |
| ITA\|PAT\|2013-11-11 | ITA\|PAT\|Nov-2013 | 98039_2013 | MW736599 | Domestic Pig | 11/11/13 | Italy | Pattada |
| ITA\|NUL\|2013-12-16 | ITA\|NUL\|Dec-2013 | 113049WB_2013 | MW736608 | Wild boar | 16/12/13 | Italy | Nulvi |
| ITA\|CAS\|2014-01-27 | ITA\|CAS\|Jan-2014 | 11484WB_2014 | SRR13975654 | Wild boar | 27/01/14 | Italy | Castelsardo |
| ITA\|VILLA\|2014-02-21 | ITA\|VILLA\|Feb-2014 | 22653/Ca/2014 | MN270980 | Domestic Pig | 21/02/14 | Italy | Villanovatulo |
| ITA\|BON\|2014-03-31 | ITA\|BON\|Mar-2014 | 35479_2014 | MW788408 | Domestic Pig | 31/03/14 | Italy | Bonorva |
| ITA\|TER\|2014-06-3 | ITA\|TER\|Jun-2014 | 51268_2014 | MW736605 | Domestic Pig | 03/06/14 | Italy | Tergu |
| ITA\|BEN\|2015-01-14 | ITA\|BEN\|Jan-2015 | 6396WB_2015 | MW736609 | Wild boar | 14/01/15 | Italy | Benetutti |
| ITA\|ORG\|2015-02-10 | ITA\|ORG\|Feb-2015 | 15998_2015 | MW736604 | Domestic Pig | 10/02/15 | Italy | Orgosolo |
| ITA\|BON\|2015-03-25 | ITA\|BON\|Mar-2015 | 28928_2015 | MW736610 | Domestic Pig | 25/03/15 | Italy | Bonorva |
| ITA\|SEN\|2015-04-10 | ITA\|SEN\|Apr-2015 | 31479_2015 | MW788407 | Domestic Pig | 10/04/15 | Italy | Sennori |
| ITA\|ANE\|2015-04-21 | ITA\|ANE\|Apr-2015 | 33747WB_2015 | MW736613 | Wild boar | 21/04/15 | Italy | Anela |
| ITA\|SAR\|2016-06-16 | ITA\|SAR\|Jun-2016 | 53706_2016 | MW736602 | Domestic Pig | 16/06/16 | Italy | Sarule |
| ITA\|DOL\|2017-01-10 | ITA\|DOL\|Jan-2017 | 3312_2017 | SRR13976564 | Domestic Pig | 10/01/17 | Italy | Dolianova |
| ITA\|DES\|2017-03-14 | ITA\|DES\|Mar-2017 | 34403WB_2017 | MW736606 | Wild boar | 14/03/17 | Italy | Desulo |
| ITA\|SEUI\|2017-05-23 | ITA\|SEUI\|May-2017 | 52060_2018 | SRR13976569 | Free Ranging Pig | 23/05/17 | Italy | Seui |
| ITA\|ARI\|2018-01-15 | ITA\|ARI\|Jan-2018 | 8343_2018 | SRR13976571 | Free Ranging Pig | 15/01/18 | Italy | Aritzo |
| ITA\|LOT\|2018-06-8 | ITA\|LOT\|1\|Jun-2018 | 54684_2018 | MW647171 | Free Ranging Pig | 08/06/18 | Italy | Lotzorai |
| ITA\|LOT\|2018-06-14 | ITA\|LOT\|2\|Jun-2018 | 56140_2018 | MW736611 | Free Ranging Pig | 14/06/18 | Italy | Lotzorai |
| ITA\|DES\|2018-06-15 | ITA\|DES\|Jun-2018 | 55234_2018 | MT932579 | Free Ranging Pig | 11/06/18 | Italy | Desulo |
| ITA\|TAL\|2018-12-15 | ITA\|TAL\|Dec-2018 | 103917_2018 | MT932578 | Free Ranging Pig | 17/12/18 | Italy | Talana |
| ITA\|LAN\|2019-01-14 | ITA\|LAN\|Jan-2019 | 7303WB_2019 | ON260839 | Wild boar | 14/01/19 | Italy | Lanusei |
| ITA\|PAT\|2019-01-14 | ITA\|PAT\|Jan-2019 | 7212WB_2019 | ON260838 | Wild boar | 13/01/19 | Italy | Pattada |
| **ITA\|BER\|2015-02-15** | **ITA\|BER\|Feb-2015** | **19155WB_2015** | **OP312970** | **Wild boar** | **15/02/15** | **Italy** | **Berchidda** |
| **ITA\|NU\|2015-04-15** | **ITA\|NU\|Apr-2015** | **33262WB_2015** | **ON260841** | **Wild boar** | **15/04/15** | **Italy** | **Nuoro** |
| **ITA\|BIT\|2016-03-16** | **ITA\|BIT\|Mar-2016** | **28784WB_2016** | **ON260840** | **Wild boar** | **16/03/16** | **Italy** | **Bitti** |
