# Supplementary material for: In vitro phenotypic characterisation of two genotype I African swine fever viruses with genomic deletion isolated from Sardinian wild boars
Source: Vet Res. 2024 Jun 7;55:73. doi: 10.1186/s13567-024-01332-8 (PMC11157848; doi:10.1186/s13567-024-01332-8)
Supplement: Supplementary file 3 — Additional file 3. Information on ASFV PCR + wild boar in Sardinia between February 2015 and January 2019. In the table information regarding geographic origin, time of collection, virological (tested organs, Ct values of real-time PCR, Malmquist) and serological (ELISA and Immunoblotting) results are reported. Samples under study are in red. [file 13567_2024_1332_MOESM3_ESM.docx]

**Additional file 3. Information on ASFV PCR^+^ wild boar in Sardinia between February 2015 to January 2019.** In the table information regarding geographic origin, time of collection, virological (tested organs, Ct values of real-time PCR, Malmquist) and serological (ELISA and Immunoblotting) results are reported. Samples under study are in red.

| Type of surveillance | ID number | Sub number | | Sample origin (location) | Month & year | Organ for virological test | RT-PCR  Ct value | Malmquist | ELISA | IB |
| --- | --- | --- | --- | --- | --- | --- | --- | --- | --- | --- |
| PASSIVE SURVEILLANCE 2015 | **33262** | **1** | | **Nuoro, loc. Monte Ortobene** | **APR 2015** | **SPLEEN** | **POS - 19.24** | **POS** |  |  |
|  |  |  |  |  |  | KIDNEY | POS - 23.63 | POS |  |  |
|  | **33747** | **1** | | **Anela, loc. Foresta** | **APR 2015** | **SPLEEN** | **POS - 15.59** | **POS** |  |  |
|  |  |  |  |  |  | KIDNEY | POS - 23.85 | POS |  |  |
| ACTIVE SURVEILLANCE 2015 | **19155** | **1** | | **Berchidda loc. Su Mulinu** | **FEB 2015** | **SPLEEN** | **POS - 17.33** | **POS** | NEG |  |
|  |  |  |  |  |  | KIDNEY | POS - 22.41 | NEG |  |  |
|  |  |  |  |  |  | LYMPHNODE | POS - 18.55 | NEG |  |  |
|  | 22048 | 7 / 8 | | Dorgali | MAR 2015 | SPLEEN | POS - 36.14 | // | POS | NEG |
|  | 29761 | 3 | | Dorgali | MAR 2015 | SPLEEN | POS - 35.74 | NEG | NEG |  |
|  | 71926 | 1 | | Alàdeisardi, S'Alduminzone | NOV 2015 | SPLEEN | POS - 29.16 | POS | N.A. |  |
|  | 72494 | 5 | | Bitti | NOV 2015 | SPLEEN | POS - 34.45 | NEG | NEG | // |
|  | 72585 | 1 | | Chiaramonti | NOV 2015 | SPLEEN | POS - 31.41 | NEG | POS | POS |
|  | 80561 | 2 | | Buddusò | NOV 2015 | SPLEEN | POS - 37.47 | NEG | POS | POS |
|  | 81505 | 3 | | Seulo | NOV 2015 | SPLEEN | POS – N.A. | NEG | NEG |  |
|  | 83018 | 3 | | Osilo | DEC 2015 | SPLEEN | POS - 37.99 | // | POS | POS |
|  | 83673 | 1 | | Bolotana | DEC 2015 | SPLEEN | POS - 37.79 | // | POS | POS |
|  | 91142 | 1 | | Buddusò | DEC 2015 | SPLEEN | POS - 36.93 | NEG | POS | POS |
|  | 94355 | 1 | | Tergu | DEC 2015 | SPLEEN | POS - 36.81 | NEG | POS | POS |
| PASSIVE SURVEILLANCE 2016 | **28784** | **1** | | **Bitti** | **MAR 2016** | KIDNEY | POS -18.30 | // |  |  |
|  |  |  |  |  |  | **SPLEEN** | **POS - 18.54** | **POS** |  |  |
|  | 30955 | 1 | | Bitti | MAR 2016 | BONE | POS - 25.82 | // |  |  |
|  | 75984 | 1 | | Desulo | OCT 2016 | SPLEEN | POS - 17.12 | NEG |  |  |
| ACTIVE SURVEILLANCE 2016 | 152 | 1 | | Bultei | JAN 2016 | SPLEEN | POS - 30.57 | NEG | N.A. |  |
|  | 3212 | 1 | | Seulo | JAN 2016 | SPLEEN | POS - 33.38 | NEG | POS | POS |
|  | 10858 | 2 | | Alàdeisardi | JAN 2016 | SPLEEN | POS - 31.86 | NEG | POS | POS |
|  | 12069 | 1 | | Arzana | JAN 2016 | SPLEEN | POS - 39.24 | NEG | POS | POS |
|  | 29061 | 1 | | Dorgali | MAR 2016 | SPLEEN | POS - 35.24 | NEG | DOUBT | NEG |
|  |  | 2 | |  |  |  | POS - 36.60 | NEG | POS | NEG |
|  |  | 3 | |  |  |  | POS - 35.70 | NEG | NEG |  |
|  |  | 5 | |  |  |  | POS - 36.12 | NEG | NEG |  |
|  | 79470 | 1 | | Buddusò, sa Contra e suzibone | NOV 2016 | SPLEEN | POS - 38.52 | NEG | POS | POS |
|  | 79519 | 5 | | Buddusò, Tazzone | NOV 2016 | SPLEEN | POS - 36.41 | NEG | NEG |  |
|  | 80193 | 1 | | Anela | NOV 2016 | SPLEEN | POS - 37.97 | NEG | NEG |  |
|  | 81534 | 3 | | Arzana, saTanca | NOV 2016 | SPLEEN | POS - 33.64 | NEG | N.E. |  |
|  | 81588 | 2 | | Bultei, sa Matta | NOV 2016 | SPLEEN | POS - 25.22 | NEG | POS | POS |
|  | 81976 | 1 | | Ozieri, San Antioco | NOV 2016 | SPLEEN | POS - 34.71 | NEG | POS | POS |
|  | 82060 | 1 | | Seui, Ardasai | NOV 2016 | SPLEEN | POS - 31.01 | NEG | POS | POS |
|  |  | 2 | |  |  |  | POS - 25.20 | NEG | NEG |  |
|  | 86476 | 3 | | Talana, suNieddumannu | NOV 2016 | SPLEEN | POS - 35.22 | NEG | POS | POS |
|  | **87152** | **1** | | **Bitti, s'Adde** | **NOV 2016** | **SPLEEN** | **POS – 30.48** | **POS** | **POS** | **POS** |
|  | **87326** | **1** | | **Padru, Scuddis** | **DEC 2016** | **SPLEEN** | **POS - 31.70** | **POS** | **POS** | **POS** |
|  | 91104 | 3 | | Arzana | DEC 2016 | SPLEEN | POS - 24.83 | NEG | POS | POS |
|  |  | 4 | |  |  |  | POS - 33.27 | NEG | POS | POS |
|  |  | 8 | |  |  |  | POS - 37,06 | NEG | POS | POS |
|  | 96536 | 1 | | Aritzo, Cossateu | DEC 2016 | SPLEEN | POS - 26,09 | // | POS | POS |
|  |  | 2 | |  |  |  | POS - 26.21 | // | POS | POS |
|  | 96623 | 1 | | Tiana, Surrrana | DEC 2016 | SPLEEN | POS - 20.50 | // | POS | NEG |
|  |  | 2 | |  |  |  | POS - 35.99 | // | NEG |  |
|  |  | 4 | |  |  |  | POS - 37.60 | // | NEG |  |
|  |  | 5 | |  |  |  | POS - 37.55 | // | NEG |  |
|  | 98987 | 3 | | Aritzo, Bidolu | DEC 2016 | SPLEEN | POS - 30.24 | NEG | POS | POS |
|  |  | 4 | |  |  |  | POS - 29.61 | NEG | POS | POS |
| PASSIVE SURVEILLANCE 2017 | **34403** | **1** | | **Desulo, Aratu** | **MAR 2017** | **SPLEEN** | **POS - 17.90** | **POS** | **NEG** |  |
|  | 36716 | 1 | | Tonara, s'ArcusuPirastru | MAR 2017 | KIDNEY | POS - 17.77 | NEG |  |  |
|  |  |  |  |  |  | SPLEEN | POS - 16.81 | NEG |  |  |
|  | 40177 | 1 | | Gadoni, Nurentulu | MAR 2017 | SPLEEN | POS - 24.43 | NEG |  |  |
|  | 84049 | 1 | | Siniscola, Zorrosca | NOV 2017 | SPLEEN | POS - 37,96 | NEG | NEG |  |
| ACTIVE SURVEILLANCE 2017 | 202 | 4 | | Padru | JAN 2017 | SPLEEN | POS - 28.58 | NEG | POS | POS |
|  | 2773 | 1 | | Buddusò, Giegheddu | JAN 2017 | SPLEEN | POS - 35.85 | // | POS | POS |
|  | 2973 | 1 | | Buddusò, Chentupojos | JAN 2017 | SPLEEN | POS - 30.07 | // | POS | POS |
|  |  | 2 | |  |  |  | POS - 38.64 | // | NEG |  |
|  | 3072 | 1 | | Arzana, Pirastreddu | JAN 2017 | SPLEEN | POS - 28.18 | NEG | POS | POS |
|  |  | 4 | |  |  |  | POS - 27.14 | NEG | POS | POS |
|  | 3085 | 3 | | VillagrandeStrisaili | JAN 2017 | SPLEEN | POS - 25.02 | NEG | // |  |
|  | 3090 | 1 | | Urzulei. Margiane e Ponte | JAN 2017 | SPLEEN | POS- 35.20 | NEG | // |  |
|  | 3092 | 3 | | Arzana, Cercecoi | JAN 2017 | SPLEEN | POS - 32.62 | NEG | POS | POS |
|  |  | 4 | |  |  |  | POS - 28.81 | NEG | // |  |
|  | 11809 | 2 | | Arzana | JAN 2017 | SPLEEN | POS - 35.35 | NEG | POS | NEG |
|  |  | 3 | |  |  |  | POS - 31.70 | NEG | POS | NEG |
|  |  | 4 | |  |  |  | POS - 31.52 | NEG | POS | NEG |
|  |  | 5 | |  |  |  | POS - 33.66 | NEG | POS | NEG |
|  | 15283 | 2 | | Pattada, Perdidu Pane | JAN 2017 | SPLEEN | POS - 36.38 | NEG |  |  |
|  | 15868 | 1 | | Seui, Trattalas | JAN 2017 | SPLEEN | POS - 34.27 | // | NEG |  |
|  | 83684 | 5 | | Oliena, Corcodde | NOV 2017 | SPLEEN | POS - 37.35 | // | NEG |  |
|  | 84114 | 1 | | Pattada, s'Olorche | NOV 2017 | SPLEEN | POS - 36.87 | // | POS | POS |
|  | 84576 | 2 | | Oliena, Marguliai | NOV 2017 | SPLEEN | POS - 23.81 | // | NEG |  |
|  |  | 3 | |  |  |  | POS - 36.72 | // | NEG |  |
|  | 84618 | 1 | | Arzana, Silose | NOV 2017 | SPLEEN | POS - 37.17 | // | POS | POS |
|  | 84705 | 1 | | Benettutti, Bodoi | NOV 2017 | SPLEEN | POS - 38.33 | // | NEG |  |
|  | 84751 | 1 | | Torpè, Filighe | NOV 2017 | SPLEEN | POS -38.51 | // | NEG |  |
|  | 89623 | 1 | | VillagrandeStrisaili, s'Iliscisuergiu | NOV 2017 | SPLEEN | POS - 38.96 | // | POS | POS |
|  | 90035 | 1 | | Ovodda | NOV 2017 | SPLEEN | POS - 38.49 | // | POS | NEG |
|  | 91792 | 1 | | Bultei, Minghinu | NOV 2017 | SPLEEN | POS - 38.44 | // | POS | POS |
|  |  | 2 | |  |  |  | POS - N.A. | // | POS | POS |
|  | 92812 | 1 | | Seulo, Lareri | NOV 2017 | SPLEEN | POS - 40.05 | // | NEG |  |
|  |  | 2 | |  |  |  | POS - 19.98 | // | NEG |  |
|  | 96698 | 1 | | NugheduS.Nicolò, Bivitì | NOV 2017 | SPLEEN | POS - 38.02 | // | POS | POS |
|  | 94535 | 1 | | Dorgali, Martaddu | DEC 2017 | SPLEEN | POS - 36.387 | // | NEG |  |
|  | 95199 | 1 | | Seulo, ConchePonti | DEC 2017 | SPLEEN | POS - 38.25 | // | POS | POS |
|  | 95555 | 1 | | Baunei, UsGiroves | DEC 2017 | SPLEEN | POS - 36.53 | // | NEG |  |
|  | 96410 | 1 | | Olzai, Nussulei | DEC 2017 | SPLEEN | POS - 37.68 | // | NEG |  |
|  | 97678 | 2 | | Aritzo, Garrei | DEC 2017 | SPLEEN | POS - 34.02 | // | POS | POS |
|  | 96744 | 1 | | Oliena, Orgoi | DEC 2017 | SPLEEN | POS - 38.05 | // | POS | POS |
|  | 100586 | 3 | | Lodè, SasPauleddasa | DEC 2017 | SPLEEN | POS - 37.22 | NEG | POS | POS |
| PASSIVE SURVEILLANCE 2018 | 46870 | 1 | | Cagliari | MAY 2018 | MUSCLE | POS - N.A. | N.A. |  |  |
| ACTIVE SURVEILLANCE 2018 | 8326 | 1 | | Villagrande Strisaili, Gerdulis | NOV 2018 | SPLEEN | POS - 36.20 | // | POS | NEG |
|  | 11232 | 7 | | Lodè, Sa Puzzonina | NOV 2018 | SPLEEN | POS - 30.38 | // | POS | NEG |
|  | 11590 | 1 | | Orosei, Santa Maria | NOV 2018 | SPLEEN | POS - 36.03 | // | NEG |  |
|  | 101629 | 1 | | Orgosolo, Olai | NOV 2018 | SPLEEN | POS - 37.92 | // | NEG |  |
| ACTIVE SURVEILLANCE 2019 | **7212** | **1** | **Pattada, Piscanu** | | **JAN 2019** | **SPLEEN** | **POS - 19.39** | **POS** | NEG |  |
|  |  |  |  |  |  | LUNG | POS - N.A. | POS |  |  |
|  | 7219 | 1 | Pattada, Michela Re | | JAN 2019 | SPLEEN | POS - 33.49 | NEG | NEG |  |
|  | **7303** | **2** | **Lanusei, Lago-Arzana-Su Pungiatu** | | **JAN 2019** | **LUNG** | **POS - 23,89** | **POS** | **POS** | **POS** |

Ct: Cycle threshold; IB: immunoblotting; //: not carried out; N.A.: data not available.

Found dead animals (passive surveillance) were not tested in ELISA; IB carried out only on ELISA+ samples.
