# Supplementary figures and images for: In vitro phenotypic characterisation of two genotype I African swine fever viruses with genomic deletion isolated from Sardinian wild boars
Source: Vet Res. 2024 Jun 7;55:73. doi: 10.1186/s13567-024-01332-8 (PMC11157848; doi:10.1186/s13567-024-01332-8)

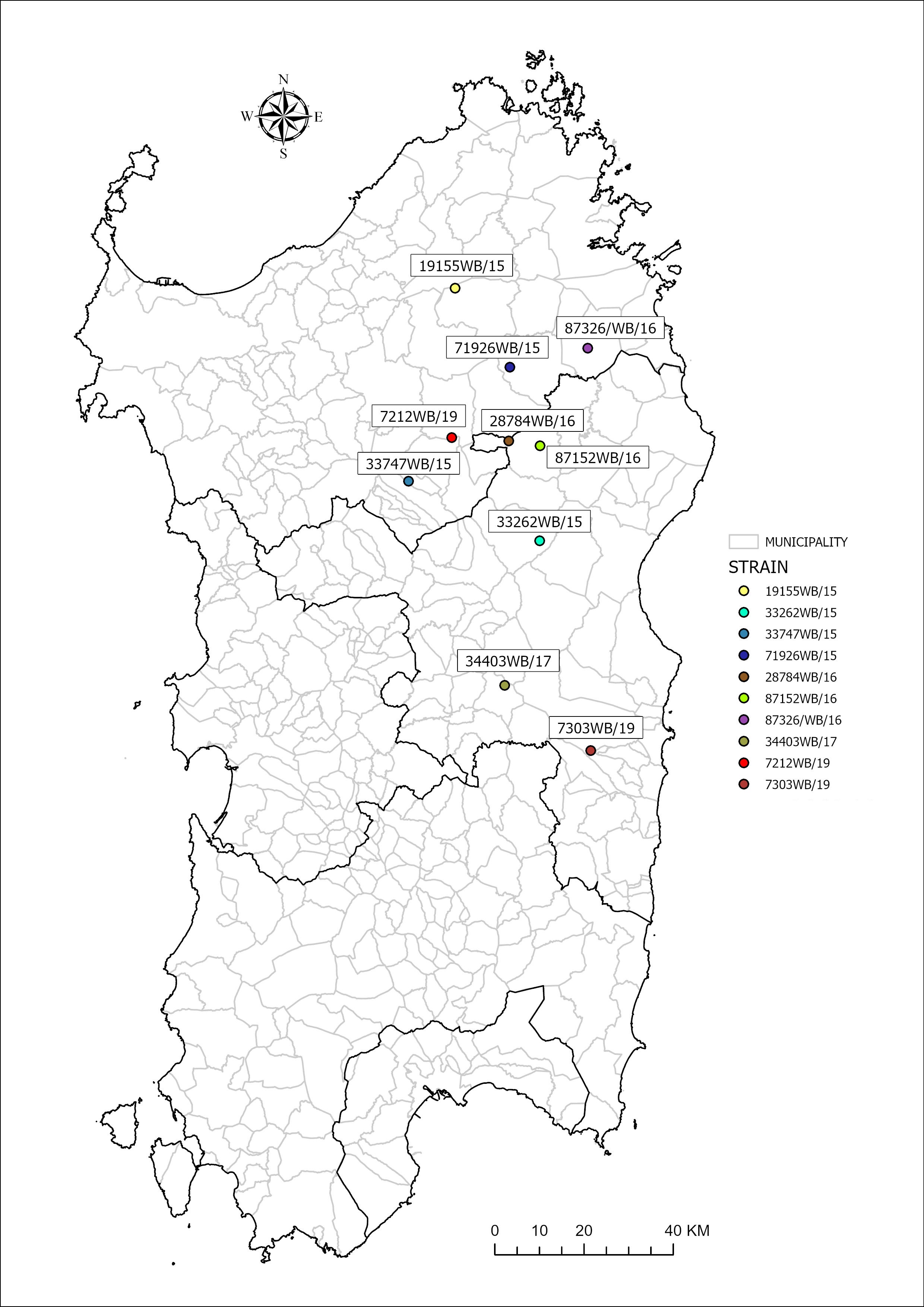

Supplement: Supplementary file 4 — Additional file 4. Map showing Sardinian ASFV wild boar isolates under study. Map showing Sardinian ASFV wild boar isolates under study. The map shows the location of the 10 wild boar isolates used in this study and collected in Sardinia between February 2015 and January 2019. [file 13567_2024_1332_MOESM4_ESM.jpg]

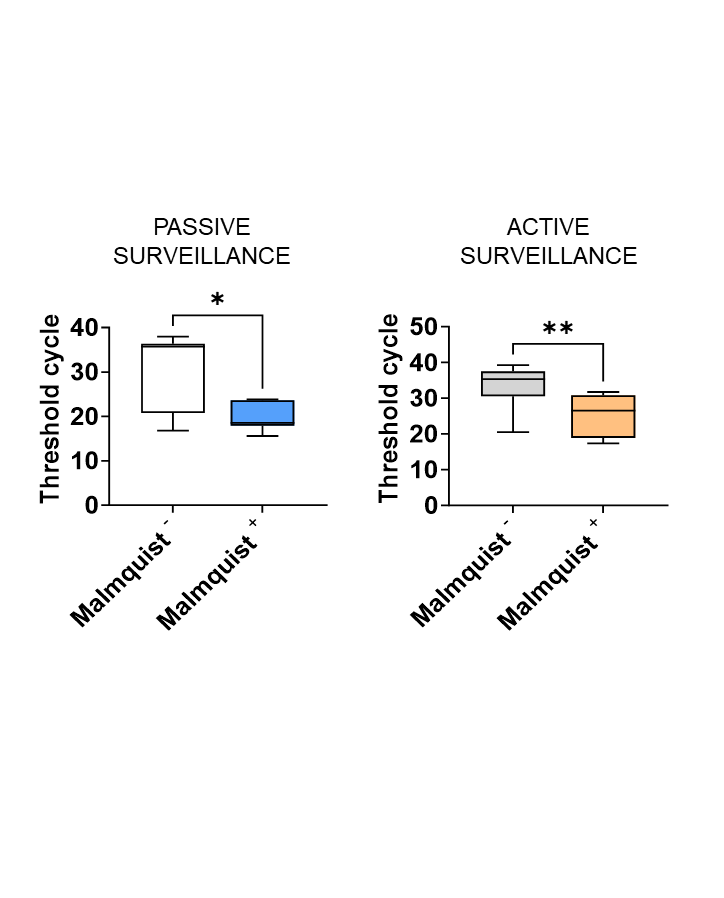

Supplement: Supplementary file 5 — Additional file 5. Threshold cycle values of real-time PCR+ wild boar samples. Wild boar isolates collected in Sardinia between February 2015 and January 2019 by both passive and active surveillance. Threshold cycle values of real-time PCR+ wild boar samples are presented. Values of Malmquist + and Malmquist- samples were compared using a Mann–Whitney test; * p < 0.05, ** p < 0.01. [file 13567_2024_1332_MOESM5_ESM.tif]

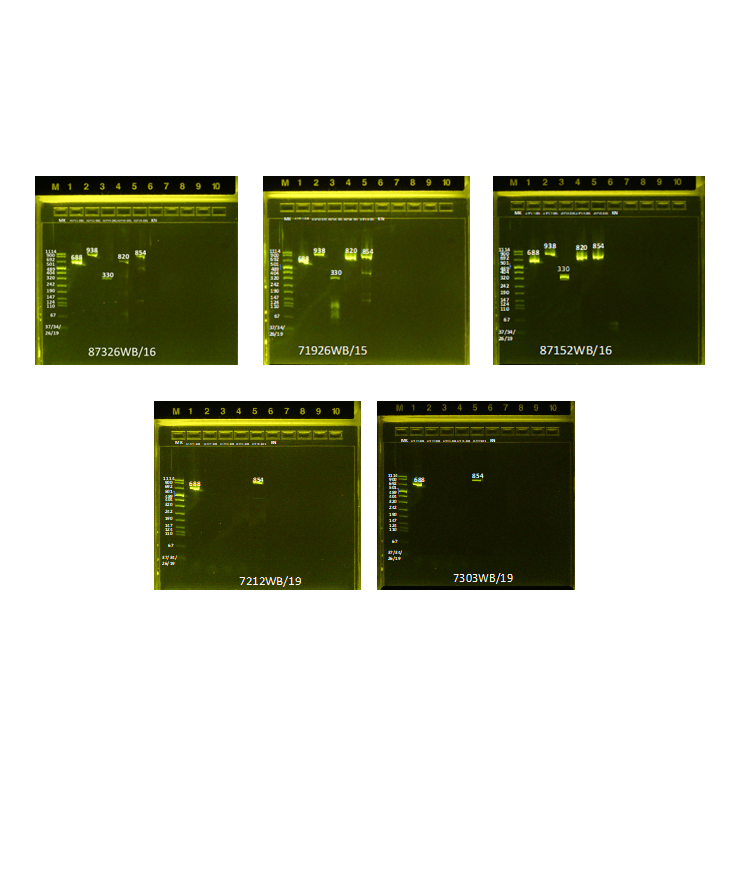

Supplement: Supplementary file 7 — Additional file 7. Agarose gel electrophoresis (2%) of PCR amplification products from five wild boar isolates under study (71926WB/15, 87152WB/16, 87326WB/16, 7303WB/19, 7212WB/19). PCR was carried out using specific primers for the deletion near the 5’ (listed in Table S1). Lane M: size marker VIII; lanes 1: ASFV1-DEL; lanes 2: ASFV2-DEL lanes 3: ASFV4-DEL lanes 4: ASFV6-DEL lanes 5: ASFV8-DEL; lane 6: K − . [file 13567_2024_1332_MOESM7_ESM.tif]
