# Supplementary material for: In vitro phenotypic characterisation of two genotype I African swine fever viruses with genomic deletion isolated from Sardinian wild boars
Source: Vet Res. 2024 Jun 7;55:73. doi: 10.1186/s13567-024-01332-8 (PMC11157848; doi:10.1186/s13567-024-01332-8)
Supplement: Supplementary file 6 — Additional file 6. Genomic sequence of the seven fully sequenced wild boar ASFV isolates collected between February 2015 and January 2019. Genomic sequence of the seven fully sequenced wild boar ASFV isolates collected between February 2015 and January 2019. [file 13567_2024_1332_MOESM6_ESM.docx]

**Additional file 6. Genomic sequence of the seven fully sequenced wild boar ASFV isolates collected between February 2015 and January 2019.**

| Strain | Size | Left # | Central | Right # | ORF * | Reference | Genbank Accession Number |
| --- | --- | --- | --- | --- | --- | --- | --- |
| 33747WB/15 | 181 753 | 40 348 | 130 241 | 11 164 | 231 | [13] | MW736613 |
| 19155WB/15 | 181 741 | 40 343 | 130 241 | 11 157 | 231 | This study | OP312970 |
| 33262WB/15 | 181 905 | 40 495 | 130 241 | 11 169 | 231 | This study | ON260841 |
| 28784WB/16 | 182 085 | 40 508 | 130 453 | 11 124 | 231 | This study | ON260840 |
| 34403WB/17 | 181 759 | 40 351 | 130 241 | 11 167 | 231 | [13] | MW736606 |
| 7303WB/19 | 177 417 | 36 006 | 130 242 | 11 169 | 228 | [14] | ON260839 |
| 7212WB/19 | 177 416 | 36 006 | 130 242 | 11 168 | 228 | [14] | ON260838 |

# Left and right refer to the left and right variable regions. The left variable region includes nucleotides from 5′-end to the beginning of A224L gene. The right variable region includes nucleotides from the end of DP238L gene to 3′ –end. Size, left, and central right are expressed in bp. * ORF, open reading frame. WB: wild boar.
